# Specific analysis of PM_2.5_-attributed disease burden in typical areas of Northwest China

**DOI:** 10.3389/fpubh.2023.1338305

**Published:** 2023-12-22

**Authors:** Qin Liao, Zhenglei Li, Yong Li, Xuan Dai, Ning Kang, Yibo Niu, Yan Tao

**Affiliations:** ^1^Key Laboratory of Western China's Environmental Systems (Ministry of Education), College of Earth and Environmental Sciences, Lanzhou University, Lanzhou, China; ^2^Northwest Institute of Eco-Environment and Resources, Chinese Academy of Sciences, Lanzhou, China; ^3^Key Laboratory of Environmental Pollution Monitoring and Disease Control, Ministry of Education, Guizhou Medical University, Guiyang, China

**Keywords:** PM_2.5_, premature mortality, specific differences, economic loss, Northwestern China

## Abstract

**Background:**

Frequent air pollution events in Northwest China pose a serious threat to human health. However, there is a lack of specific differences assessment in PM_2.5_-related disease burden. Therefore, we aimed to estimate the PM_2.5_-related premature deaths and health economic losses in this typical northwest region, taking into account disease-specific, age-specific, and region-specific factors.

**Methods:**

We utilized the WRF-Chem model to simulate and analyze the characteristics and exposure levels of PM_2.5_ pollution in Gansu Province, a typical region of Northwest China. Subsequently, we estimated the premature mortality and health economic losses associated with PM_2.5_ by combining the Global Exposure Mortality Model (GEMM) and the Value of a Statistical Life (VSL).

**Results:**

The results suggested that the PM_2.5_ concentrations in Gansu Province in 2019 varied spatially, with a decrease from north to south. The number of non-accidental deaths attributable to PM_2.5_ pollution was estimated to be 14,224 (95% CI: 11,716–16,689), accounting for 8.6% of the total number of deaths. The PM_2.5_-related health economic loss amounted to 28.66 (95% CI: 23.61–33.63) billion yuan, equivalent to 3.3% of the regional gross domestic product (GDP) in 2019. Ischemic heart disease (IHD) and stroke were the leading causes of PM_2.5_-attributed deaths, contributing to 50.6% of the total. Older adult individuals aged 60 and above accounted for over 80% of all age-related disease deaths. Lanzhou had a higher number of attributable deaths and health economic losses compared to other regions. Although the number of PM_2.5_-attributed deaths was lower in the Hexi Corridor region, the per capita health economic loss was higher.

**Conclusion:**

Gansu Province exhibits distinct regional characteristics in terms of PM2.5 pollution as well as disease- and age-specific health burdens. This highlights the significance of implementing tailored measures that are specific to local conditions to mitigate the health risks and economic ramifications associated with PM_2.5_ pollution.

## 1 Introduction

Air pollution, particularly fine particulate matter (PM_2.5_), is the fourth leading determinant of mortality worldwide (1). Epidemiological studies have shown that long-term exposure to ambient PM_2.5_ can lead to adverse health outcomes, including increased risks of death from disease such as ischemic heart disease (IHD), stroke, chronic obstructive pulmonary disease (COPD), lower respiratory infections (LRI), and lung cancer (LC) (2–5). In the last decade, PM_2.5_ has become the predominant air pollutant in China. Although there has been a significant reduction in PM_2.5_ levels (6, 7), the majority (81%) of the population is still exposed to annual average PM_2.5_ concentration that exceed the World Health Organization's (WHO) Air Quality Interim Target of 35 μg·m^−3^ (8). According to estimates from the Global Burden of Disease Study (GBD), PM_2.5_ pollution resulted in ~4.14 million premature deaths worldwide in 2019, with over 1/4 of these deaths occurring in China, a notably higher number than in other countries (1). The health impacts of PM_2.5_ also result in significant economic losses to society (9, 10). Guan et al. (10) estimated that the economic losses from ambient PM_2.5_ pollution were 3.20–3.34 trillion yuan across China during 2015–2017. Therefore, a diligent and accurate evaluation of the disease burden caused by PM_2.5_ pollution remains essential for effective policy formulation.

Many studies have utilized ground monitoring data to assess the premature mortality attributable to PM_2.5_ (11–13). However, accurately understanding the characteristics of PM_2.5_ pollution poses challenges due to the limited spatial coverage and uneven distribution of the PM_2.5_ monitoring network (14). Methods of PM_2.5_ exposure based on air quality model simulations with broader spatial coverage are considered more effective (15). For example, Wu et al. (16) and Li et al. (17) estimated PM_2.5_-related premature mortality in China using PM_2.5_ concentration data simulated by air quality models. Furthermore, the exposure-response function is crucial for accurately assessing premature mortality. Recent studies have recognized a non-linear relationship in the relative risk of PM_2.5_ health effects (3, 4). As a result, a progression of exposure-response models have been developed, ranging from basic linear models to more advanced log-linear models (LL), integrated exposure-response model (IER), and the most recent global exposure mortality model (GEMM) (5).

Several studies have examined the national-level impact of PM_2.5_ exposure on disease burden using varying PM_2.5_ concentration data and exposure-response functions (6, 7, 18–20). However, there has been less focus on the disease burden of PM_2.5_ in specific regions, and it is mainly done in developed regions such as the Beijing-Tianjin-Hebei region (21), the Yangtze River Delta (22), and the Pearl River Delta (23), while researches in the northwestern region of China are lacking. PM_2.5_-related mortality varies significantly across regions due to disparities in air pollution levels and socio-economic status (24). Additionally, age structure plays a significant role in PM_2.5_-related mortality, as different diseases and age groups contribute variably to these deaths. Xie et al. (25) found that overlooking age structure could result in an overestimation of premature deaths by 14%. Reports on the variations in PM_2.5_-related mortality across different age groups are limited (7, 26). Hence, it is imperative to comprehensively estimate the specific impact of PM_2.5_-related mortality on different diseases and age groups within specific regions.

**Figure 1:**
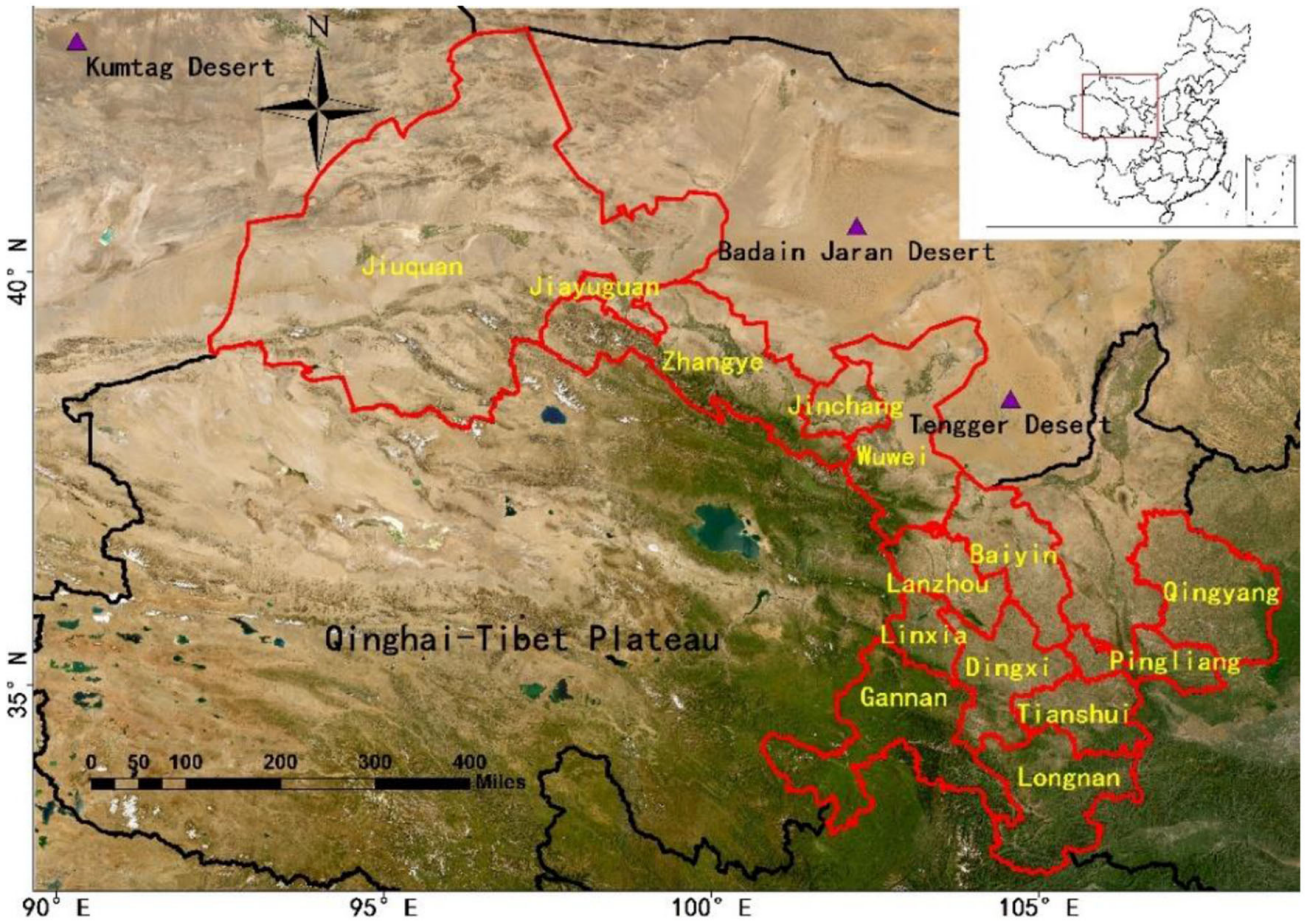
Geographical location of the study area.

Gansu Province, located in the northwest of China, is a typical underdeveloped area. It is situated at the convergence of the Loess Plateau, Qinghai-Tibet Plateau, and Inner Mongolia Plateau, making it prone to sand and dust storms (27) (Figure 1). The region is affected by both human activities and natural sources of particulate pollution. However, the extent of the disease burden caused by PM_2.5_ pollution in Gansu Province is not clear. To address this knowledge gap, this study aims to assess PM_2.5_-related mortality and its associated health economic losses in Gansu Province in 2019 using the WRF-Chem air quality model in combination with the optimized GEMM model. Additionally, the study quantifies the specific differences in premature deaths across different diseases, ages, and regions. The ultimate goal is to provide a scientific basis for the development of effective measures to reduce the health impacts of air pollution.

## 2 Methodology

### 2.1 Simulations of PM_2.5_ concentration

The WRF-Chem air quality model was employed to estimate PM_2.5_ concentrations in Gansu Province for the year 2019. The simulation domain covered the entire Gansu Province and its surrounding provinces, with a horizontal resolution of 20 × 20 km (150 × 100 grids). The meteorological initial conditions were derived from the 6-h National Centers for Environmental Prediction (NCEP) final analysis data, with a spatial resolution of 1° × 1° (28). The initial conditions for atmospheric chemistry were obtained from the Community Atmosphere Model with Chemistry (CAM-Chem) model, with a 6-h interval and a spatial resolution of 1.9° × 2.5° (29). Anthropogenic emissions data was sourced from the Multi-resolution Emission Inventory for China (MEIC) developed by Tsinghua University (30). Biomass burning emissions were derived from the Fire INventory from NCAR (FINN) (31). Biogenic emissions were based on the commonly used Model of Emissions of Gases and Aerosols from Nature (MEGAN) inventory (32). Dust emissions were implemented using the Air Force Weather Agency (AFWA) emission scheme (33). The selected physical and chemical parameterization schemes for the simulation are presented in Table 1.

**Table 1:** Main physical and chemical parameters adopted in the WRF-Chem simulation.

**Type**	**Scheme**	**Parameter**
Physical options	Boundary layer scheme	Mellor-Yamada Nakanishi and Niino 2.5
	Microphysics process scheme	Morrison 2-moment
	Radiation scheme	Rapid radiative transfer model for GCM
	Land surface process scheme	Noah
	Cumulus parameterization scheme	Grell-3D
Chemical options	Gas-phase chemical mechanism	Model for ozone and related tracers
	Aerosol module	Model for simulating aerosol interactions and chemistry with 4 sectional bins
	Photolysis reaction	Fast troposphere ultraviolet visible (F-TUV)

The WRF-Chem model simulation results were evaluated using environmental monitoring data. The daily average PM_2.5_ concentration data for Gansu Province in 2019 were sourced from the Gansu Provincial Environmental Monitoring Center Station. These data covered 33 national air quality monitoring sites across 14 cities and prefectures. Various evaluation metrics were used, including normalized mean bias (NMB), normalized mean error (NME), mean fractional bias (MFB), mean fractional error (MFE), and the correlation coefficient (R). Overall, the simulation of PM_2.5_ concentrations in Gansu Province in 2019 showed good performance (34, 35). The NMB, NME, MFB, and MFE values were 0.08, 0.29, 0.04, and 0.20, respectively, and the results of *R* (0.56) was significant at the 1% level (*P* < 0.01).

### 2.2 Calculation of premature mortality

The Global Exposure Mortality Model (GEMM) optimized by Burnett et al. (5) was employed to estimate premature mortality attributable to PM_2.5_ pollution in adults (aged 25+). The GEMM took into account deaths from non-communicable diseases and lower respiratory infections (NCD+LRI), which are considered as non-accidental deaths. It also considers deaths from five major diseases, namely IHD, stroke, COPD, LC, and LRI, which represent deaths caused by specific diseases. The difference between non-accidental deaths and the sum of deaths from these five specific diseases represents deaths from other diseases. The computation formula used is as follows:


(1) $$\begin{array}{l} M_{i,j}=Pop\times PS_{j}\times B_{i,j}\times \frac{\left(RR_{i,j}-1\right)}{RR_{i,j}} \end{array}$$

(2) $$RR_{i,j}=\{\begin{array}{c} \exp\left\{\frac{\theta_{i,j}\log\left(\frac{C-C_{0}}{\alpha_{i,j}}+1\right)}{1+exp\left(-\frac{C-C_{0}-\mu_{i,j}}{\nu_{i,j}}\right)}\right\},if\;C>C_{0}{\;}_{i,j}\\ \;1,\;if\;C\leq C_{0}{\;}_{i,j} \end{array}$$

where the subscripts *i* and *j* represent the disease type and age structure (25–29, 30–34, 35–39, 40–44, 45–49, 50–54, 55–59, 60–64, 65–69, 70–74, 75–79 and ≥80 years old), respectively; *M*_*i,j*_ is premature mortality caused by PM_2.5_ exposure; *Pop* refers to the exposed population to PM_2.5_; *PS*_*j*_ is the proportion of a specific age group within the exposed population; *B*_*i,j*_ represents the baseline mortality rate; *RR*_*i,j*_ is the relative risk; *C* is the annual average PM_2.5_ concentration; *C*_0_ is the counter-factual concentration below which it is assumed that there is no additional risk (2.4 μg·m^−3^) (1, 18); θ, α, μ, and ν are fitting parameters for the PM_2.5_ exposure-response function. The values for θ, α, μ, and ν can be found in the references provided by Burnett et al. (5). Population data was sourced from the Gansu Development Yearbook 2020 (36). Age structure data and baseline mortality rates for each age group come from the China Cause-of-Death Surveillance Dataset 2019 (37), with data from the western region applied to Gansu Province.

Considering the uncertainty of RR in the model, a 95% confidence interval (CI) was calculated using the standard error in the GEMM:


(3) $$95\%\;CI\;(RR_{i,j})=\exp\left\{\frac{\left(\theta_{i,j}\pm 1.96\times SE\left(\theta_{i,j}\right)\right)\times log\left(\frac{C-C_{0}}{\alpha_{i,j}}+1\right)}{1+exp\left(-\frac{C-C_{0}-\mu_{i,j}}{\nu_{i,j}}\right)}\right\}$$

where *SE(*θ_*i,j*_*)* represents the standard deviation of θ_*i,j*_, with its value referenced in the study by Burnett et al. (5).

### 2.3 Evaluation of health economic loss

The VSL was used to assess the economic losses resulting from PM_2.5_-related premature deaths. VSL quantifies the monetary value individuals are willing to pay (WTP) to reduce the death risk and is commonly used in assessing health economic losses related to air pollution (13, 38). The formula is as follows:


(4) $$\begin{array}{l} EB_{g,t}=M_{i,j}\times VSL_{g,t} \end{array}$$

where *EB*_*g,t*_ represents the health economic losses in region *g* (i.e., Gansu Province) in year *t* attributed to PM_2.5_. *VSL*_*g,t*_ indicates the VSL in Gansu Province in year *t*. Since specific VSL results for Gansu Province are not available, this study adopts the VSL survey results from existing domestic regions as a reference. The benefit transfer method is employed, adjusting for differences in per capita GDP across different regions and timeframes. The formula is as follows:


(5) $$\begin{array}{l} VSL_{g,t}=VSL_{b}\times {\left(\frac{GDP_{g}}{GDP_{b}}\right)}^{\eta }\times {\left(1+\Delta P_{g}+\Delta G_{g}\right)}^{\eta } \end{array}$$

where *VSL*_*b*_ represents the VSL of the reference region. For this study, we have selected the latest VSL survey results for Beijing in 2016 conducted by Jin et al. (39), which amount to 5.54 million yuan. *GDP*_*g*_ and *GDP*_*b*_ represent the per capita GDP of Gansu Province and Beijing in 2016, respectively. η is the income elasticity of VSL, and we have adopted the recommended value of 0.8 from the Organization for Economic Co-operation and Development (OECD) (40). Δ*P*_*g*_ is the percentage change in the Consumer Price Index (CPI) in year *t* for Gansu Province compared to 2016. Δ*G*_*g*_ is the percentage change in per capita GDP in year *t* for Gansu Province compared to 2016. The per capita GDP and CPI for Gansu Province in 2019 are sourced from the Gansu Development Yearbook 2020 (36), while the per capita GDP for Beijing in 2016 come from the China Statistical Yearbook 2017 (41).

## 3 Results

### 3.1 PM_2.5_ pollution characteristics

**Figure 2:**
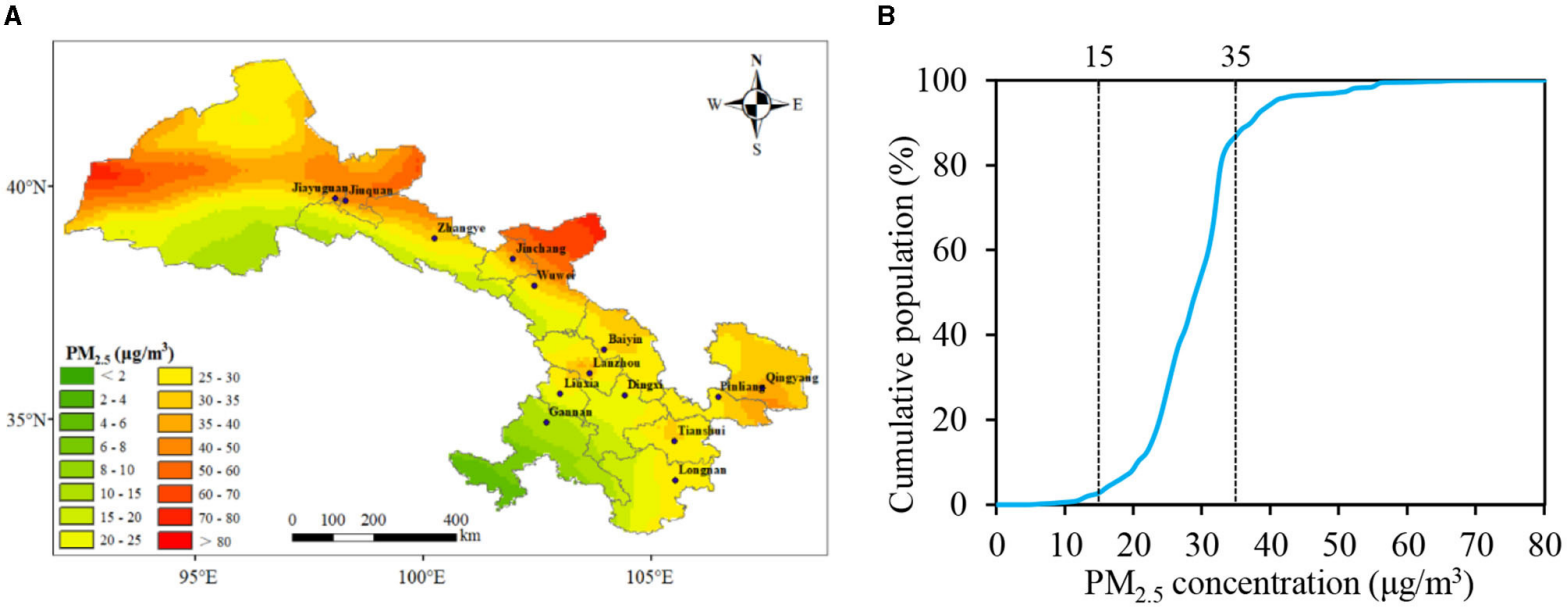
Exposure levels of PM_2.5_ in Gansu Province, 2019. **(A)** Spatial distribution of PM_2.5_ concentration; **(B)** cumulative distribution in various levels of PM_2.5_ concentration.

Based on the WRF-Chem simulation data, the spatial distribution of the annual average PM_2.5_ concentration in Gansu Province in 2019 is shown in Figure 2. The overall distribution exhibited higher concentrations in the north and lower concentrations in the south. The regions with higher concentrations were mainly located in the Hexi Corridor region and certain parts of the central-eastern region. Specifically, Jiuquan and Jiayuguan recorded the highest population-weighted annual mean PM_2.5_ concentrations, reaching 41.48 and 40.28 μg·m^−3^, respectively, exceeding the Chinese Ambient Air Quality Standards (CAAQS) (35 μg·m^−3^ for Grade II). Qingyang, Wuwei, and Jinchang followed closely, with concentrations ranging between 32.82 and 34.86 μg·m^−3^. The concentrations in Lanzhou, Pingliang, Baiyin, and Zhangye all exceeded 30 μg·m^−3^. Gannan registered the lowest concentration at 12.22 μg·m^−3^. Notably, the vast majority of areas in Gansu Province had an annual average PM_2.5_ concentration exceeding 15 μg·m^−3^ for Grade I in CAAQS.

Using the simulated PM_2.5_ concentration and population data, we calculated the cumulative distribution of the population under different PM_2.5_ concentrations for 2019 (Figure 2). It can be observed that in 2019, 86.6% of the population in Gansu Province lived in areas with an annual average PM_2.5_ concentration below 35 μg·m^−3^. However, only 2.7% of the population resided in areas where the PM_2.5_ concentration was smaller than 15 μg·m^−3^.

### 3.2 Cause-specific premature mortality

**Figure 3:**
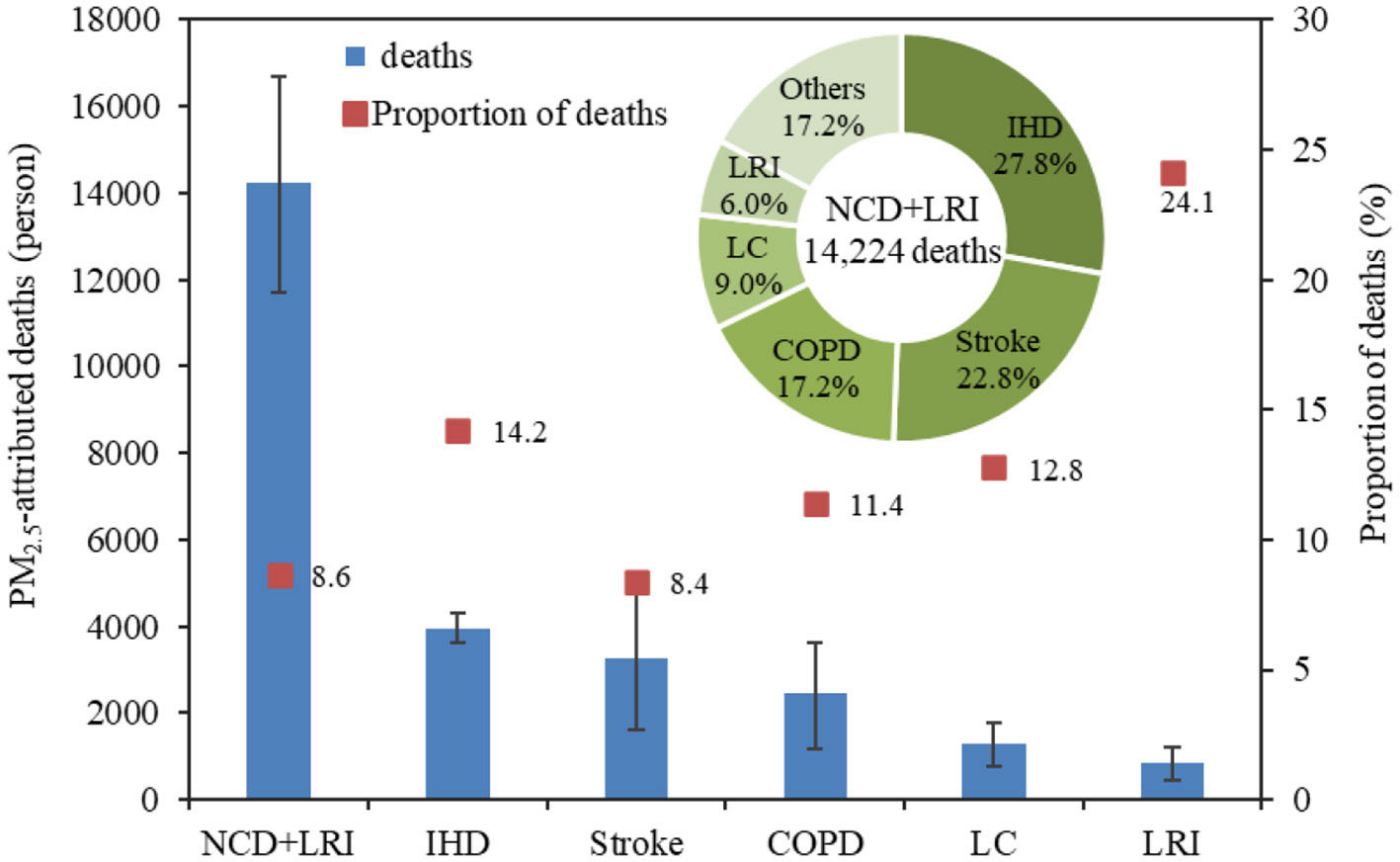
The number and proportion of PM_2.5_-attributed deaths in Gansu Province, 2019.

Using the GEMM model, we estimated the mortality burden attributable to PM_2.5_ pollution in Gansu Province in 2019 (Figure 3). According to the GEMM NCD+LRI model, there were 14,224 (95% CI: 11,716–16,689) non-accidental deaths due to PM_2.5_ pollution in Gansu Province in 2019, accounting for 8.6% of the total deaths. The numbers of PM_2.5_-attributed deaths for IHD, stroke, LC, COPD, and LRI were 3,956 (95% CI: 3,608–4,299), 3,244 (95% CI: 1,602–4,807), 2,440 (95% CI: 1,189–3,615), 1,286 (95% CI: 780–1,764), and 853 (95% CI: 445–1,204) respectively, and represented 14.2, 8.4, 11.4, 12.8, and 24.1% of the deaths from the corresponding specific causes. It was evident that around a quarter of LRI deaths were caused by PM_2.5_ pollution, followed by IHD. Meanwhile, <1/10 of stroke deaths could be attributed to PM_2.5_. Although LRI deaths were more closely associated with PM_2.5_ pollution, the absolute number of deaths from LRI was much lower than those from IHD and stroke due to its lower baseline mortality rate.

When examining the proportion of deaths attributable to PM_2.5_ for different diseases relative to non-accidental deaths (NCD+LRI), the proportion for IHD was the highest at 27.8%, followed by stroke at 22.8%, and the combined percentage of these two diseases accounted for more than 50%. COPD, LC, and LRI constituted 17.2, 9.0, and 6.0%, respectively, while deaths from other diseases made up 17.2%. From this, it can be inferred that the majority of PM_2.5_-attributed deaths come from IHD and stroke. Moreover, a substantial proportion is due to causes other than these five specific diseases.

### 3.3 Age-specific premature mortality

**Figure 4:**
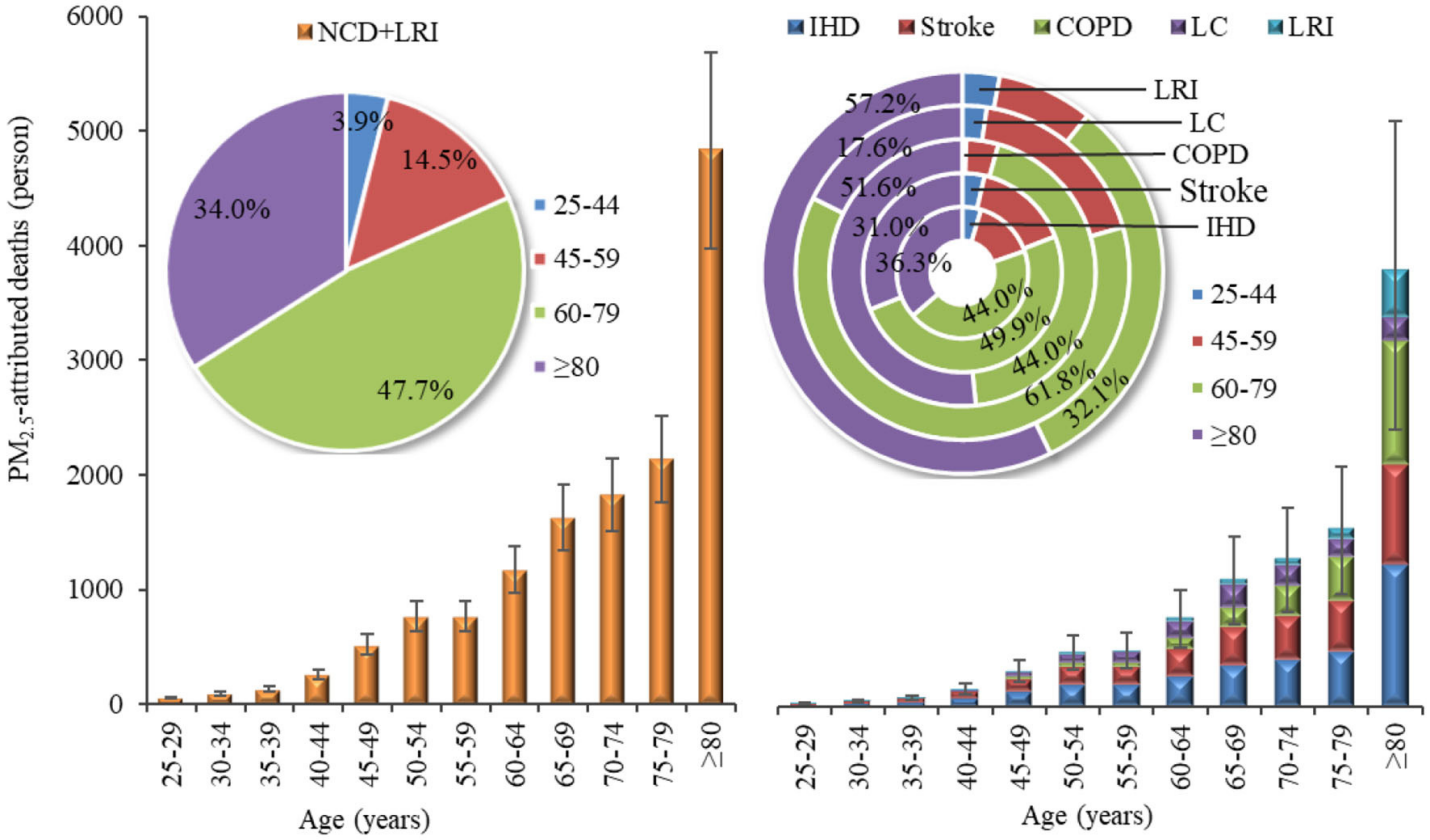
PM_2.5_-attributed deaths by age group in Gansu Province, 2019.

Figure 4 present the number and proportion of deaths attributable to PM_2.5_ pollution by age group in Gansu Province in 2019. It was obvious that there were substantial differences in the number of deaths from various diseases caused by PM_2.5_ across different age groups. Generally, the number of non-accidental deaths and disease-specific deaths attributable to PM_2.5_ increased with age. There were 11,615 (95% CI: 9,562–13,633) non-accidental deaths in people aged 60 and above, representing 81.7% of all non-accidental deaths, which was much higher than that of people under 60 years old. Notably, 34.0% of these deaths were reported in the age group of 80 and above.

IHD was the primary cause of death burden across all age groups. For those under the age of 80, the number of deaths from stroke exceeded that from COPD, whereas for those aged 80 and above, deaths due to COPD outnumbered those from stroke. The age distribution of IHD and stroke deaths attributable to PM_2.5_ pollution mirrored the patterns seen with non-accidental deaths. Among those aged 60 and older, the numbers of IHD and stroke deaths were 3,178 (95% CI: 2,896–3,457) and 2,625 (95% CI: 1,292–3,901), respectively, accounting for 80.3 and 80.9% of the total deaths from these diseases across all age groups. For the same age bracket (aged 60+), the numbers of COPD and LRI deaths attributable to PM_2.5_ were 2,334 (95% CI: 1,137–3,457) and 762 (95% CI: 398–1,075), respectively, representing a staggering 95.6 and 89.3% of the total deaths from these conditions across all ages. Within this, the contribution from those aged 80 and above alone exceeded half, at 51.6 and 57.2%, respectively. For LC deaths attributable to PM_2.5_ across all age strata, the highest numbers were still among those aged 60 and above, with 1,021 (95% CI: 620–1,400) deaths, constituting 79.4% of all LC deaths. It was worth noting that, unlike other diseases, the proportion of LC deaths was highest in the 60–74 age group (17.9%) and those aged 80 and above (17.6%).

### 3.4 Region-specific premature mortality

**Figure 5:**
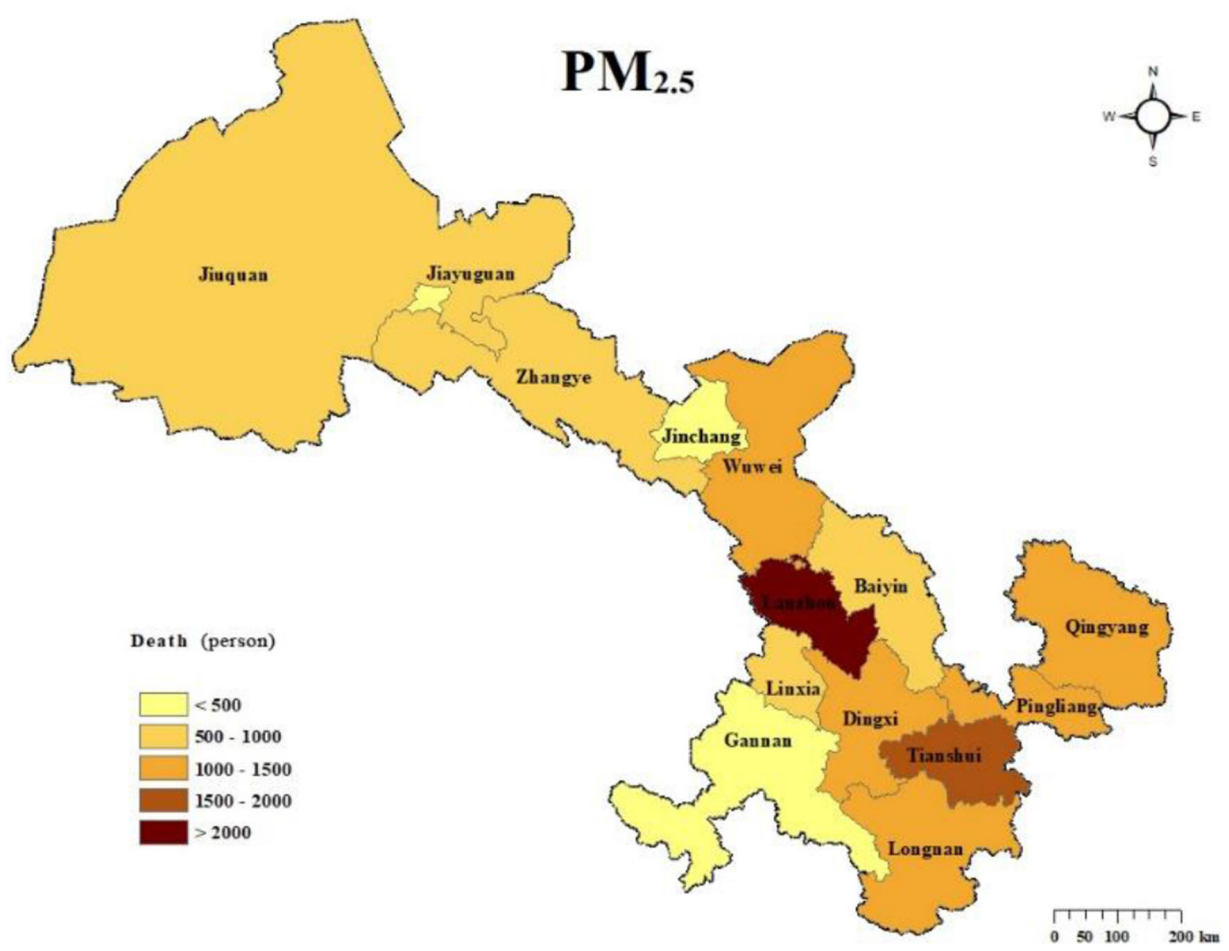
Spatial distribution of PM_2.5_-attributed deaths in Gansu Province, 2019.

The spatial distribution of non-accidental deaths attributable to PM_2.5_ pollution in Gansu Province in 2019 is illustrated in Figure 5. Lanzhou, the provincial capital, recorded the highest number of PM_2.5_-attributed deaths at 2,103 (95% CI: 1,733–2,467), accounting for 15.0% of the total non-accidental deaths in the province. Tianshui followed with 1,757 (95% CI: 1,447–2,062) deaths, making up 12.5% of the provincial total. The cities of Qingyang, Dingxi, Longnan, Pingliang, and Wuwei reported attributed death numbers ranging between 1,000 and 1,500. Jiayuguan, Gannan, and Jinchang, on the other hand, had lower non-accidental death counts, all under 500. It was observed that areas with higher population densities also exhibited higher numbers of non-accidental deaths attributable to PM_2.5_ pollution. Although the Hexi Corridor region had relatively high PM_2.5_ concentrations, the lower population density of this area, especially in Jiayuguan and Jinchang, resulted in significantly fewer PM_2.5_-attributed deaths compared to other regions.

### 3.5 Health economic loss

**Figure 6:**
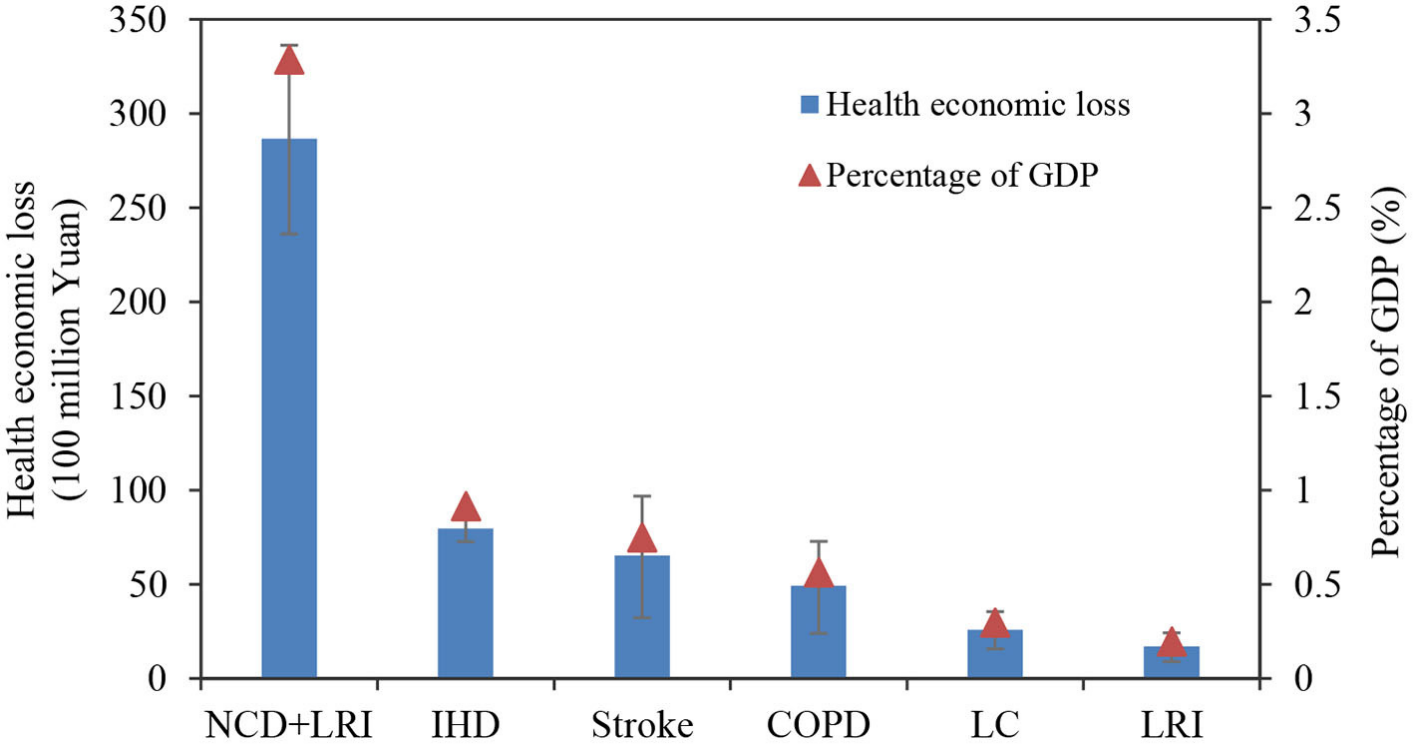
Health economic loss attributable to PM_2.5_ pollution in Gansu Province in 2019.

Based on the assessment of deaths attributable to PM_2.5_, the health economic loss associated with PM_2.5_-attributed mortality in Gansu Province was estimated using the VSL method, as depicted in Figure 6. In 2019, the health economic loss caused by PM_2.5_ in Gansu Province amounted to 28.66 (95% CI: 23.61–33.63) billion yuan, accounting for 3.3% of the region's GDP. The combined health economic losses for the five diseases were calculated to be 23.74 (95% CI: 15.36–31.61) billion yuan.

**Figure 7:**
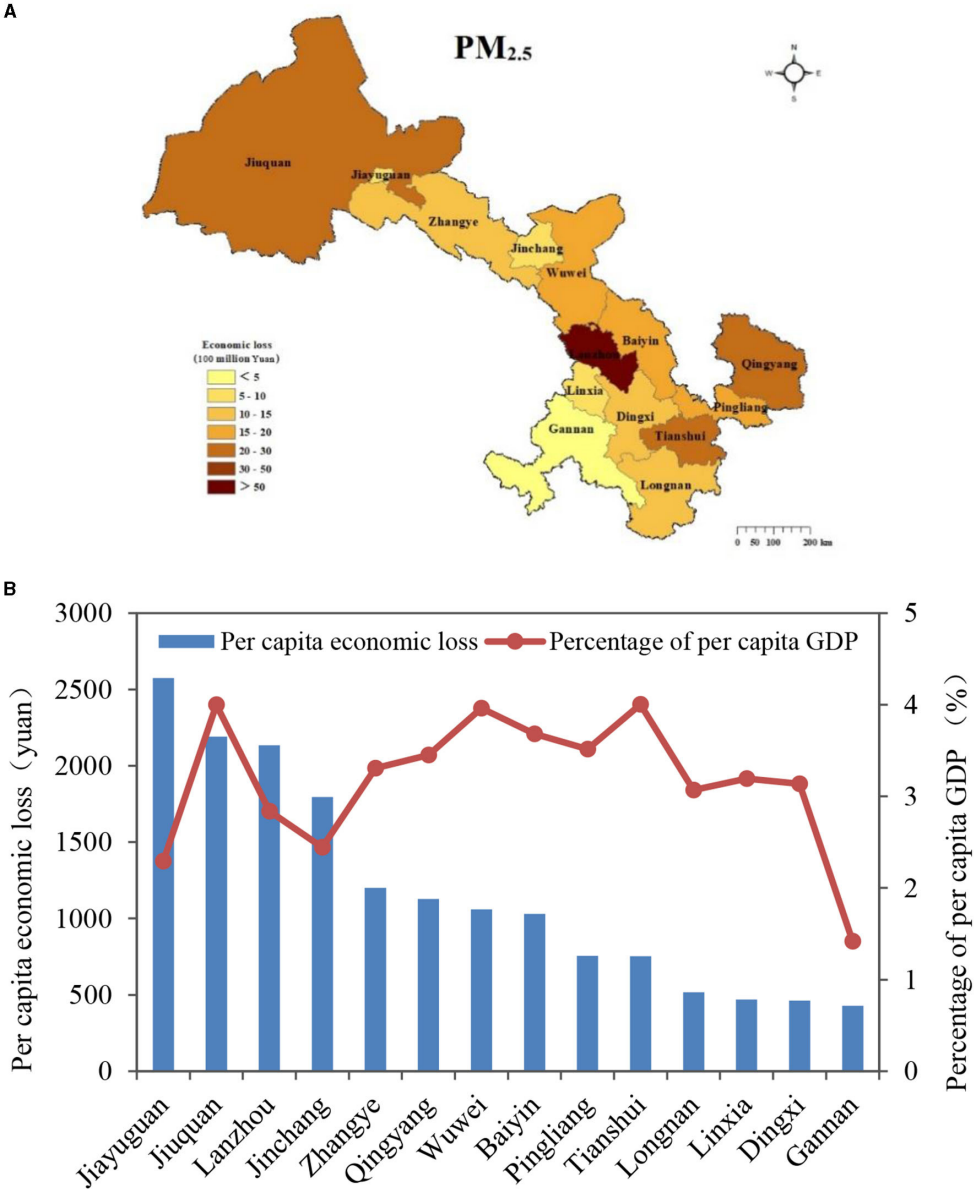
Health economic losses attributable to PM_2.5_ pollution in Gansu Province in 2019. **(A)** Spatial distribution of health economic losses; **(B)** per capita health economic losses and the proportion of per capita GDP.

For the various regions (Figure 7), Lanzhou experienced the highest health economic loss, totaling 8.10 (95% CI: 6.67–9.50) billion yuan, contributing 29.0% to the overall health economic loss in Gansu Province. This was followed by Qingyang, Tianshui, and Jiuquan, whose combined contributions accounted for 27.2% of the total health economic loss in the province. Gannan reported the lowest health economic loss at 0.31 (95% CI: 0.25–0.37) billion yuan. The per capita health economic losses caused by PM_2.5_ across various regions in Gansu Province ranged from 428 to 2,575 yuan. Jiayuguan recorded the highest per capita health economic loss, reaching 2,575 yuan. Jiuquan and Lanzhou followed closely with per capita losses of 2,190 and 2,135 yuan, respectively, while Jinchang also experienced a relatively high per capita loss of 1,796 yuan. Meanwhile, the ratio of per capita health economic loss to per capita GDP revealed that Jiuquan, Tianshui, Wuwei, and Baiyin had notably high proportions. Conversely, although Jiayuguan and Jinchang had elevated per capita health economic losses, their ratios in relation to per capita GDP were lower. Moreover, Gannan exhibited the lowest figures both in terms of per capita health economic loss and its proportion to per capita GDP.

## 4 Discussion

The spatial distribution of PM_2.5_ concentrations in Gansu Province varied significantly due to the differences in sources and emission levels of air pollutants. The Hexi region, situated in the western dust corridor of China, was severely influenced by the Tengger Desert, Badain Jaran Desert, and Kumtag Desert (42). Sandstorms had the most effect on Jiuquan and Jiayuguan, located in the westernmost part of Gansu Province (27). In the central and eastern regions of Gansu Province, cities like Lanzhou, Qingyang, Pingliang, and Baiyin had higher air pollutant emissions from anthropogenic sources such as industry and transportation. Conversely, in the southern areas, Gannan and Longnan relied more on green industries like eco-tourism and agricultural product processing, resulting in lower total air pollutant emissions (43). In conclusion, the PM_2.5_ concentration in Gansu Province was influenced by both natural and anthropogenic sources. Therefore, it is crucial for Gansu Province, located in the northwest of China, to consider the multiple sources of PM_2.5_ and implement region-specific measures to address PM_2.5_ pollution.

The study results showed the number of cardiovascular diseases (IHD and stroke: 7,200 individuals) deaths attributable to PM_2.5_ pollution in Gansu Province was higher than respiratory diseases (COPD, LC, and LRI: 4,579 individuals) deaths. This finding is consistent with previous research conducted in other regions of China (16, 26, 44). The higher number of premature cardiovascular deaths can be attributed to the generally high baseline mortality rate from cardiovascular diseases (45). In a study conducted in 2019, it was also found that IHD and stroke were the primary causes of PM_2.5_-attributed mortality in China (25). However, the proportion of COPD and LRI-related deaths (15.8%) was lower compared to ours (23.2%), possibly due to higher baseline mortality rates for these diseases in western China. According to the China Cause-of-Death Surveillance Dataset 2019 (37), the baseline mortality rates for COPD and LRI in western China were notably higher than in the central and eastern regions. Some studies have also observed that while PM_2.5_-attributed deaths from respiratory diseases have been decreasing, deaths from cardiovascular diseases (especially IHD) have been increasing (17). This trend is expected to continue with the rise of unhealthy lifestyles and an aging population. Therefore, in addition to reducing PM_2.5_ pollution levels, it is important to focus on improving healthcare and promoting healthier lifestyles to lower the baseline mortality rate of cardiovascular diseases and reduce the number of PM_2.5_-related deaths from such conditions in the future.

**Figure 8:**
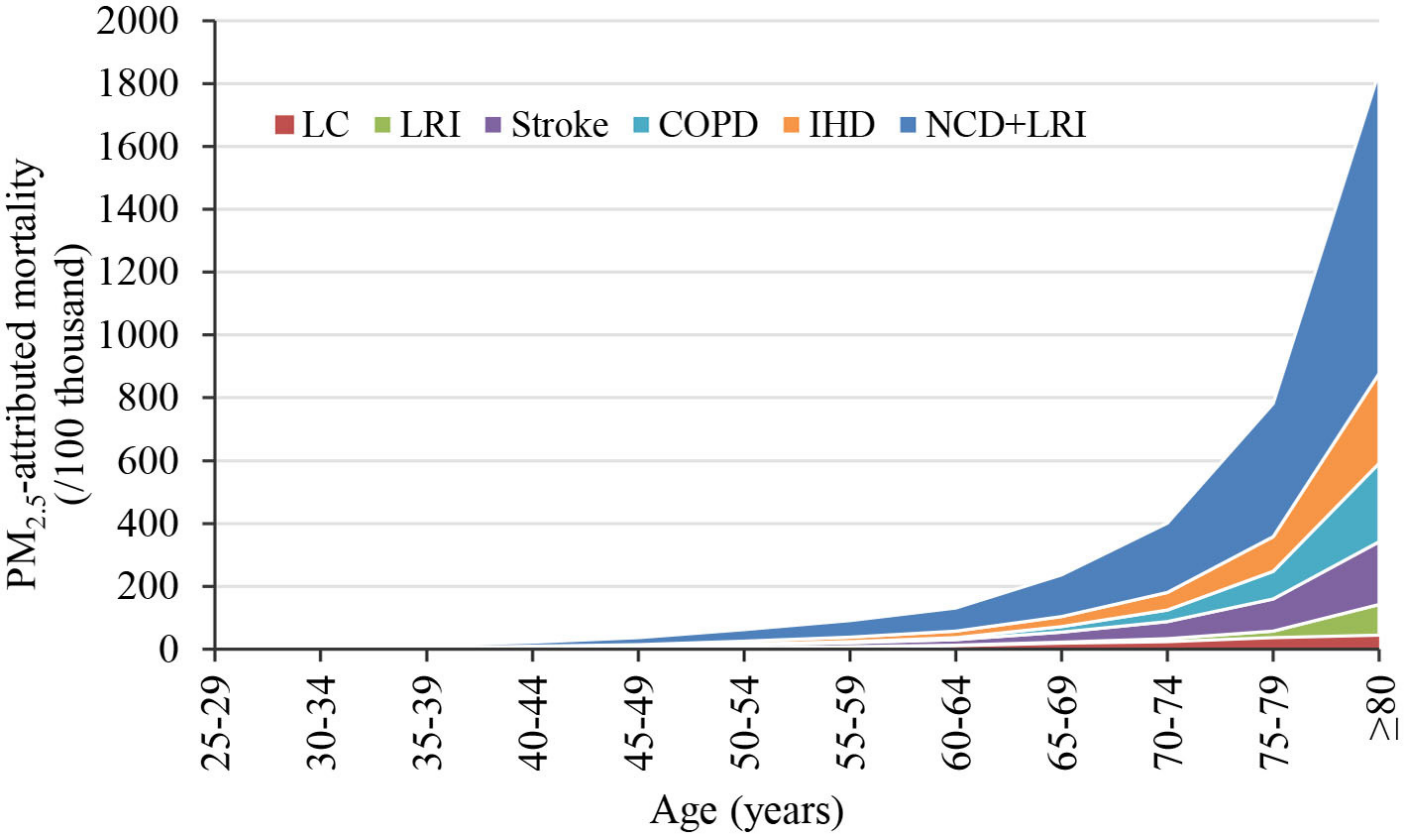
PM_2.5_-attributed mortality rates for different age groups in Gansu Province, 2019.

Considering the variations in total population among different age groups, this study further calculated the PM_2.5_-attributed mortality rate to better compare the premature deaths caused by PM_2.5_ pollution in different age categories (Figure 8). It was evident that the PM_2.5_-attributed mortality rate showed substantial disparities among different age groups, with a noticeable increase in older age groups. The non-accidental mortality attributable to PM_2.5_ reached 960 per 100,000 individuals in the population aged 80 and above. This finding is consistent with previous studies (46), indicating that older adults are more susceptible to the adverse effects of air pollution due to their elevated baseline mortality rate (16). Therefore, it is essential to consider the impacts of air pollution on different age groups and diseases and implement proactive and effective measures to shield older adults and enhance their overall health. Additionally, given the projected increase in the aging population in the future (47), it is imperative to estimate the influence of age structure on mortality attributed to air pollution in order to accurately understand the health effects on the population.

Premature deaths attributable to PM_2.5_ varied significantly across different regions, with the number of deaths being mainly influenced by PM_2.5_ concentration and population density when using a consistent baseline mortality rate. Lanzhou, a provincial capital, had a higher number of PM_2.5_-attributed deaths compared to other regions. This can be attributed to the combination of sandstorms from the Hexi Corridor and surrounding areas, rapid economic growth, heavy industries, and transportation development in Lanzhou. As a result, Lanzhou has elevated PM_2.5_ concentrations and the highest population density, leading to significant health implications. Research by Guan et al. (48) revealed that air pollution-related health impacts in regional hub cities contribute significantly to the overall health burden within the province, especially in central and western China. Although Jiayuguan, an industrially advanced city, has higher PM_2.5_ concentrations, its unique population distribution with low population density results in a lower health impact. The distribution of PM_2.5_-related health economic losses in different regions shows similarities, but there are still some disparities. These variations primarily stem from health economic losses being dependent on the level of attributable deaths and health costs, denoted as the value of VSL, which is influenced by the local level of economic development (13, 49). Even if the per capita health economic loss is relatively low, it can still represent a higher proportion of the per capita GDP. In other words, the economic burden caused by air pollution can be considerable.

Despite the important findings outlined above, there are still significant uncertainties and limitations in estimating the disease burden attributable to PM_2.5_ pollution. First, different means of measuring PM_2.5_ may affect the concentration data. Simulation results with the WRF-Chem model faced uncertainties from emission inventories and simulations of chemical-physical processes. We adopted anthropogenic emission data from MEIC, which has been widely used in air quality simulation. Meanwhile, the reliability of the WRF-Chem model simulation was evaluated using common evaluation metrics. Second, the exposure-response relationship between PM_2.5_ and health outcomes was also a major source of uncertainty (44). Previous studies mainly utilized the IER model, which only incorporated cohort study data from European and American regions, potentially underestimating the health burden in areas with higher PM_2.5_ concentrations. In contrast, the GEMM model considered higher air pollution levels and included the results of a cohort study in China, estimating a 120% larger mortality burden than the IER model (5). Therefore, it may be more appropriate to use exposure-response models based on specific Chinese cohort studies, although the accuracy of the GEMM model requires further scientific validation (26, 50). Third, the exposure-response model assumes that the toxicity of ambient PM_2.5_ is only influenced by concentrations, but the health effects of PM_2.5_ from different chemical components or different sources may vary greatly (38). This is particularly important in Gansu Province, which has complex PM_2.5_ emission sources and lacks relative risk functions for specific sources. Fourth, due to more elaborate data limitations, this study used baseline mortality rates and age structure from western China for Gansu Province, and did not consider their spatial variability across the study region, which may have introduced some discrepancies in the estimated results. Fifth, the VSL played a key role but also caused uncertainties when assessing health economic losses. The VSL estimates from developed countries could not be applied to China due to differences in socio-economic characteristics and air pollution levels. There are relatively few studies on VSL conducted in China (39, 51–53). However, owing to differences in the timing of willingness-to-pay surveys and economic development levels, the values of VSL were considerable uncertainties, leading to significant variations in the estimated health economic losses. In view of the rising income level in China in recent years and the increasing public awareness of air pollution, the results of the more recent VSL survey were used in our study.

## 5 Conclusion

This study utilized simulated PM_2.5_ concentration data and an exposure-response model to investigate the impact of PM_2.5_ pollution on premature deaths and health economic losses in Gansu Province. The results indicated that there were 14,224 non-accidental deaths attributed to PM_2.5_ pollution in 2019, with the majority caused by IHD and stroke. Older adults (aged 60+) were more affected by PM_2.5_ pollution than those under 60 years old. The distribution of deaths varied spatially, with high concentrations in densely populated areas like Lanzhou and Tianshui. The health economic losses due to PM_2.5_ pollution accounted for 3.3% of the annual GDP, with Lanzhou contributing the most. Jiayuguan, Jiuquan, and Lanzhou had higher per capita health economic losses. In conclusion, there are significant differences in the diseases, age groups, and regional distribution of disease burden attributable to PM_2.5_ in Gansu Province. It is recommended to implement region-specific measures to address PM_2.5_ pollution and improve the health of older adults to prevent more deaths and economic losses.

## Data availability statement

The original contributions presented in the study are included in the article/supplementary material, further inquiries can be directed to the corresponding author.

## Author contributions

QL: Conceptualization, Methodology, Writing—original draft. ZL: Formal analysis, Writing—original draft. YL: Methodology, Visualization, Writing—review & editing. NK: Formal analysis, Writing—review & editing. XD: Formal analysis, Writing—review & editing. YN: Visualization, Writing—original draft. YT: Conceptualization, Writing—review & editing.
